# Acute stroke requires sophisticated and demanding management to achieve an optimal outcome

**DOI:** 10.51866/lte.879

**Published:** 2026-04-21

**Authors:** Sounira Mehri, Josef Finsterer

**Affiliations:** 1 Biochemistry Laboratory, LR12ES05 "Nutrition-Functional Foods and Vascular Health", Faculty of Medicine, Monastir, Tunisia.; 2 Neurology Neurophysiology Center, Vienna, Austria.

**Keywords:** Ischaemic stroke, Risk factors, Magnetic resonance imaging, Cognitive impairment, Depression

**Dear Editor,**

We read with interest the article by Ahmad et al. about a 42-year-old man who experienced a transient ischaemic attack (TIA) and, a week later, multiple lacunar infarcts that presented clinically as dysarthria and left-sided haemiparesis.^1^ After returning to work, he showed cognitive deficits that required cognitive rehabilitation.^1^ The authors concluded that tailored interventions are needed in primary stroke care to optimise cognitive recovery and improve overall quality of life.^1^ The study is noteworthy, but several points should be discussed.

The first point is that there was no mention of how the presence of mild cognitive impairment (MCI) was ruled out in the index patient even before the stroke. It was unclear whether there were any indications of MCI through history-taking with the relatives, friends or work colleagues of the index patient.

The second point is that the article did not mention whether diabetes, hyperlipidaemia and arterial hypertension were well or poorly controlled at the time of lacunar stroke in 2022.^1^ Although blood pressure was described as normal on admission, the HbAlc levels, 24-hour blood pressure monitoring outcomes and serum lipid levels should have been specified.

The third point is that cardiac causes for the stroke were not adequately excluded. The article did not report whether the index patient had atrial fibrillation on admission or during hospitalisation. No echocardiography results were provided to determine whether the patient had systolic dysfunction, endocarditis, Takotsubo syndrome, left ventricular noncompaction or intraventricular thrombus formation. Given the presence of multiple risk factors for myocardial infarction, it would also have been important to rule out coronary artery disease.

The fourth point is that MCI was diagnosed using the Montreal Cognitive Assessment (MoCA) rather than a comprehensive neuropsychological test battery.^1^ The index patient should have been evaluated by a specialised neuropsychologist before and after rehabilitation to assess the cognitive modalities that were impaired, the degree of the impairments and the success of the rehabilitation in terms of MCI.^2^

The fifth point is that the term ‘haemiplegic gait’ was misleading. Paralysis means the complete loss of motor function (i.e. the inability to move a limb or tense a muscle). If the leg muscles are paralysed on one side, the patient cannot walk at all. He might be able to stand on one leg, but he would not be able to walk without support. The contradiction between the description of the clinical examination as ‘haemiparesis’ and the description of ‘haemiplegic gait’ should have been resolved.

The sixth point is that post-stroke depression could not be adequately ruled out in the index patient. Depression is a common complication of serious illness in general and particularly after stroke and is usually amenable to antidepressant treatment.^3^

The seventh point is that the term ‘recent stroke’ was not defined.^1^ The authors should have specified whether they meant treatment of a TIA 1 week before the stroke, the patient’s first stroke in his life, a juvenile insult or an acute stroke. If they meant an acute stroke, it should have been stated whether the patient received thrombolysis, thrombectomy or both. The time that elapsed between the onset of stroke symptoms and arrival at the hospital should also have been specified. In addition, it should have been clarified whether the stroke was classified as acute or subacute.

The eighth point refers to the limitation of the study that no cerebral magnetic resonance imaging (MRI) scan was provided.^1^ To assess in which vascular territory and in which dimension the stroke occurred, the authors should have presented a corresponding image with the relevant modalities used for the diagnosis of an ischaemic stroke. The age of the stroke can be assessed more reliably with multimodal MRI than with cerebral CT. The results of the CT or MR angiography of the intra- and extracranial arteries were also lacking.

The ninth point is that the authors did not report how post-stroke depression was appropriately ruled out as a differential diagnosis for stroke-related MCI. They should have specified which depression scale was used to determine whether post-stroke depression was present.

The tenth point is that no further cerebral imaging was performed after the manifestation of MCI. Repeating cerebral imaging would have been useful to rule out the possibility that MCI represented a recurrence of stroke.

In summary, this interesting study has limitations that put the results and their interpretation into perspective. Addressing these limitations could strengthen the conclusions and improve the overall interpretation of the study. All open questions should be clarified before readers uncritically accept the conclusions of the study. The management of acute stroke requires sophisticated and comprehensive diagnostics and care in both prehospital and inpatient settings to determine as early as possible whether a patient is a candidate for thrombolysis or thrombectomy. Before stroke- associated MCI is diagnosed, stroke recurrence and post-stroke depression should be ruled out.
